# Primary membranous glomerulonephritis with negative serum PLA2R in haemophilia A successfully managed with rituximab – case report and review of the literature

**DOI:** 10.1186/s12882-021-02475-y

**Published:** 2021-07-22

**Authors:** Nicholas Meyer, Wendy Cooper, Paul Kirwan, Roger Garsia, Scott Dunkley, David M. Gracey

**Affiliations:** 1 Department of Renal Medicine, Royal Prince Alfred Hospital, Camperdown, NSW Australia; 2 Tissue Pathology and Diagnostic Oncology, NSW Health Pathology, Royal Prince Alfred Hospital, Camperdown, NSW Australia; 3 Central Clinical School, Faculty of Medicine, University of Sydney, Sydney, NSW Australia; 4 School of Medicine, Western Sydney University, Campbelltown, NSW Australia; 5 Electron Microscopy Unit, Department of Anatomical Pathology, Concord Repatriation General Hospital, Concord, NSW Australia; 6 Department of Immunology, Royal Prince Alfred Hospital, Camperdown, NSW Australia; 7 Institute of Haematology, Royal Prince Alfred Hospital, Camperdown, NSW Australia

**Keywords:** PLA2R, Membranous nephropathy, Glomerulonephritis, Haemophilia, Rituximab

## Abstract

**Background:**

Hepatitis C virus (HCV) and human immunodeficiency virus (HIV) cause a wide range of glomerular pathologies. In people with haemophilia, transfusion-associated infections with these viruses are common and definitive pathological diagnosis in this population is complicated by the difficulty of safely obtaining a renal biopsy. Membranous nephropathy (MN) is a common cause of adult onset nephrotic syndrome occurring in both primary and secondary forms. Primary MN is associated with podocyte autoantibodies, predominantly against phospholipase A2 receptor (PLA2R). Secondary disease is often associated with viral infection; however, infrequently with HIV or HCV. Distinguishing these entities from each other and other viral glomerular disease is vital as treatment strategies are disparate.

**Case presentation:**

We present the case of a 48-year-old man with moderate haemophilia A and well-controlled transfusion-associated HCV and HIV coinfection who presented with sudden onset nephrotic range proteinuria. Renal biopsy demonstrated grade two membranous nephropathy with associated negative serum PLA2R testing. Light and electron microscopic appearances were indeterminant of a primary or secondary cause. Given his extremely stable co-morbidities, treatment with rituximab and subsequent angiotensin receptor blockade was initiated for suspected primary MN and the patient had sustained resolution in proteinuria over the following 18 months. Subsequent testing demonstrated PLA2R positive glomerular immunohistochemistry despite multiple negative serum results.

**Conclusions:**

Pursuing histological diagnosis is important in complex cases of MN as the treatment strategies between primary and secondary vary significantly. Serum PLA2R testing alone may be insufficient in the presence of multiple potential causes of secondary MN.

## Background

In people with haemophilia, renal disease secondary to infection with human immunodeficiency virus (HIV) or hepatitis C virus (HCV) is common, with rates approaching 95% in some cohorts [1, 2]. Nephrotic syndrome in this population, therefore, carries a broad differential; however, definitive diagnosis is challenging given the bleeding risk associated with renal biopsy [3]. Distinguishing between HIV associated nephropathy (HIVAN), HCV associated glomerulonephritis (GN) and the de novo onset of a new GN is vital given vastly disparate therapeutic approaches. Several cases of successful renal biopsy in people with haemophilia via both transjugular [4] and percutaneous [5, 6] approaches have been reported.

MN is a common cause of GN in adults and is pathologically characterised by diffuse thickening of the glomerular basement membrane (GBM) and subepithelial deposition of immune complexes [7]. Primary (or idiopathic) MN is associated with autoantibodies against podocyte proteins. These include either phospholipase A2 receptor (PLA2R) or thrombospondin type-1 domain containing 7A (THSD7A) in 70–80% of patients and more rarely neural epidermal growth factor-like 1 (NELL-1) and Semaphorin 3b [8–10]. Secondary membranous nephropathy is associated with immune complex deposition in association with systemic lupus erythematosus, HIV [11], HCV [12, 13], IgG4 disease [14] and various drugs. Primary and secondary MN may also be histologically distinct; primary is more likely to be associated with IgG4 deposition compared with other subclasses [15] and less likely to have mesangial immune complex deposits [16].

Treatment of primary MN is initially supportive; however, immunosuppressive therapy is recommended in those who do not respond to conservative measures [17]. The recent MENTOR trial [18] has shown rituximab as a useful first-line therapy. Alternate treatment approaches may involve calcineurin inhibitors [19, 20] or cyclophosphamide [21], often paired with glucocorticoids. The role of rituximab in secondary MN remains unclear as most trials excluded this cohort. We present a case demonstrating the diagnostic challenges associated distinguishing primary from secondary MN in the presence of multiple comorbid conditions.

## Case presentation

A 48-year-old man with moderate haemophilia A, transfusion-associated HIV and previously successfully treated transfusion-associated HCV with cirrhosis was referred to the outpatient renal department for evaluation of new onset proteinuria. His HIV was contracted from transfusion over 20 years prior and was well controlled on maraviroc/efavirenz/raltegravir with a CD4^+^ count of 1200 cells/mm^3^ and an undetectable viral load. He had completed treatment for genotype 1A HCV with ledipasvir/sofosbuvir 2 years prior to his presentation. There were mild associated portal hypertensive changes with splenomegaly, oesophageal varices and a mild thrombocytopaenia of 106 × 10^9^/L. There was no family or personal history or renal disease. He was asymptomatic, though had noted a one-year history of foamy urine. Physical examination was notable only for palpable splenomegaly without evidence of peripheral oedema.

Outpatient urinary investigations revealed a 24-h urine protein excretion of 4.35 g, with urine microscopy demonstrating 24 × 10^6^ red cells. The serum creatine was unremarkable at 98 μmol/L (1.10 mg/dL) and serum albumin of 51 g/L (5.1 g/dL). Serological investigations showed anti-nuclear antibody (ANA) was mildly elevated at 1:160 with a homogenous pattern. Anti-neutrophil cytoplasmic antibody (ANCA), double stranded DNA and anti-GBM antibodies were negative, as was testing for serum anti-PLA2R and cryoglobulins. Serum protein electrophoresis did not demonstrate any monoclonal bands. Ultrasonography of the kidneys demonstrated bilateral normal size with unremarkable corticomedullary differentiation and no evidence of hydronephrosis. There were bilateral simple renal cysts without evidence of renal calculi.

**Fig. 1:**
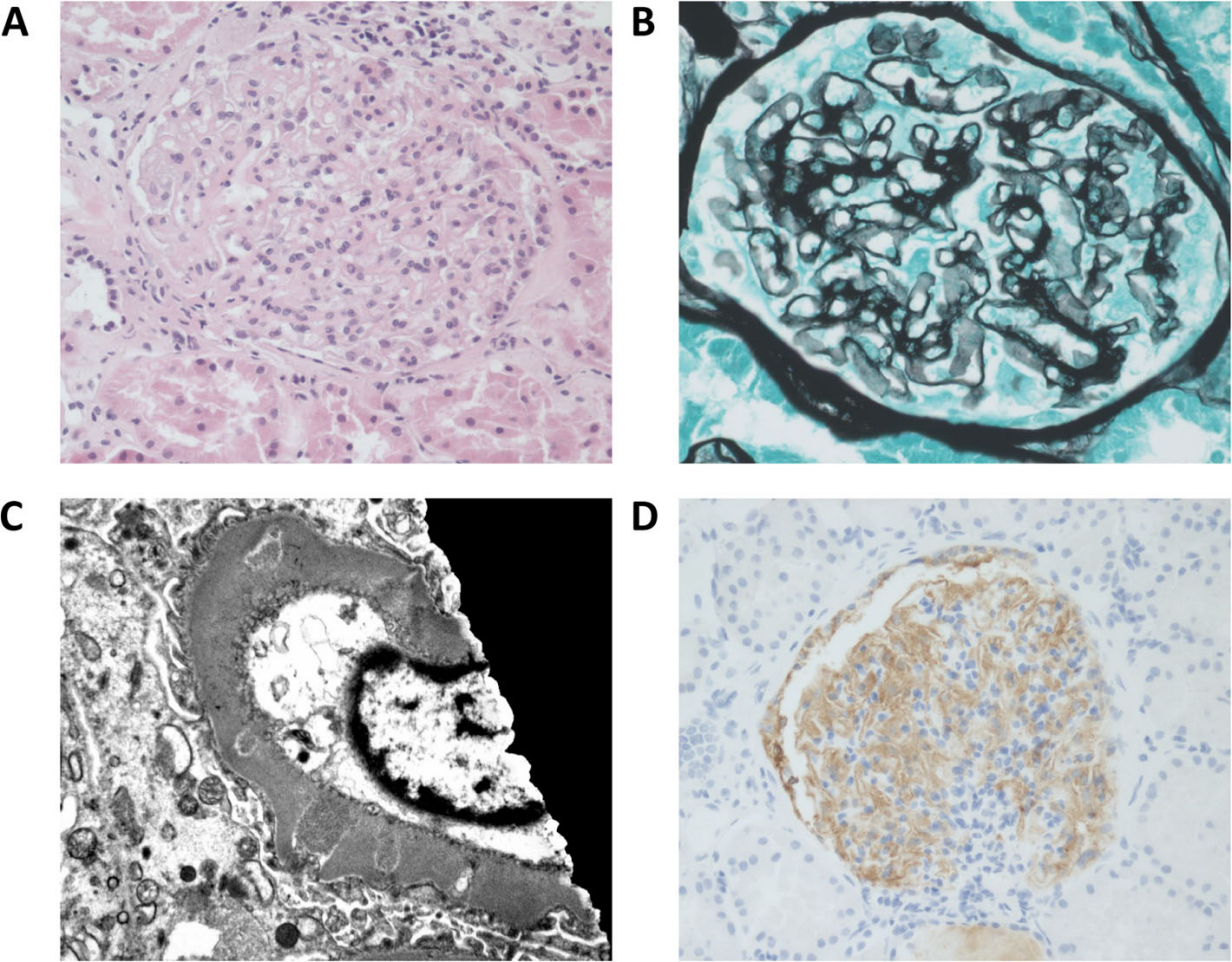
Renal biopsy findings. **A** Haematoxylin and eosin staining demonstrates mild mesangial expansion and hypercellularity. **B** Silver staining demonstrates altered capillary loops with vacuolisation of the basement membrane. **C** Electron micrograph with scattered dense and lucent deposits in a membranous pattern. **D** Immunohistochemistry for anti-PLA2R with positive capillary loop staining

An inpatient admission was organised to facilitate a renal biopsy with appropriate factor and platelet coverage given a factor VIII level of 3% prior to the procedure. 4000 IU of recombinant factor VIII was administered and percutaneous renal biopsy was performed unremarkably under ultrasound guidance. 3000 IU further factor VIII was given at 12 and 24 h post biopsy and no bleeding complications were observed. Light microscopy demonstrated enlarged glomeruli with very mild mesangial expansion and hypercellularity (Fig. 1A). A silver stain showed abnormal glomerular capillary loops with vacuolisation of the basement membrane (Fig. 1B). There were no crescents or necrotising lesions and Congo red staining for amyloid was negative. Immunofluorescence demonstrated patchy, nonspecific IgM, IgA, C1q and fibrinogen staining with distinct peritubular staining for C3 but no staining was identified in glomerular capillary loops. IgG could not be assessed due to a lack of residual tissue and the high clinical risk of obtaining further tissue for biopsy. Electron micrographs showed normal BM thickness with a few small dense and lucent deposits in a membranous pattern consistent with stage 2 membranous nephropathy (Fig. 1C) [7]. Further examination for secondary causes of MN were undertaken. Hepatic ultrasound demonstrated no evidence of hepatocellular carcinoma and there was no evidence of other associated malignant disease. HIV viral load and HCV RNA remained undetectable.

**Fig. 2:**
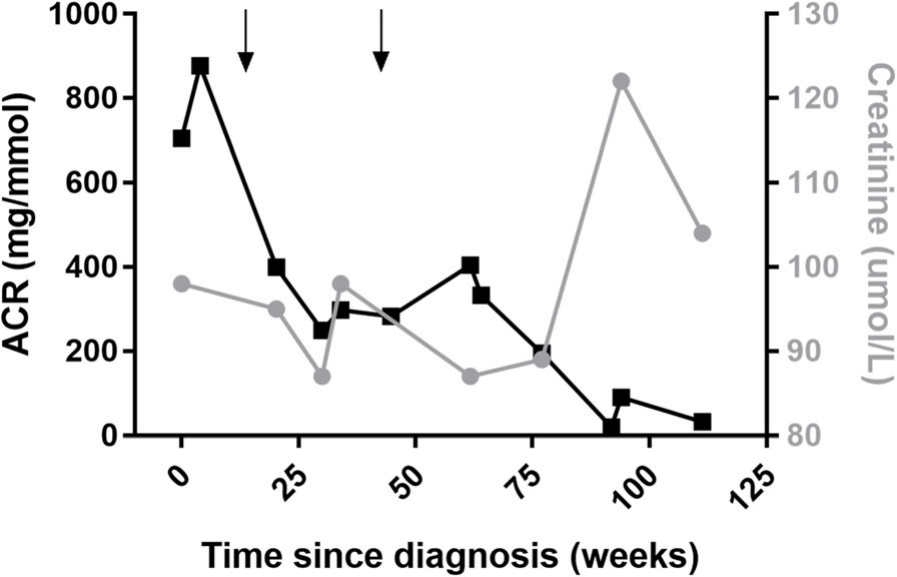
Response to treatment. Albumin to creatinine ratio (left axis) and serum creatinine (right axis) versus weeks since diagnosis of MN. Arrows indicate the administration of a full cycle of rituximab (two 1000 mg doses separated by 2 weeks)

Despite negative serum testing for PLA2R, in the absence of a clear secondary cause of MN, a trial of rituximab was initiated. Two doses of 1000 mg rituximab were administered intravenously 2 weeks apart. Angiotensin receptor blockade with candesartan was commenced after the initial response to rituximab and subsequent redosing occurred at 6 months. Throughout this time, he remained asymptomatic. Subsequently, the original renal biopsy specimens underwent anti-PLA2R immunohistochemical staining (that was not available at the time of original diagnosis), which demonstrated positive glomerular capillary loop staining. (Fig. 1D) Serial measurements of ACR along over the following 18 months showed a steady reduction to a nadir of 32.1 mg/mmol and serum creatinine was stable throughout this period (Fig. 2). Serial serum anti-PLA2R testing over this period has remained negative and there has been ongoing sustained virological response of both HIV and HCV despite immunosuppressive therapy.

## Discussion and conclusions

To our knowledge, this is the first reported case of membranous glomerulonephritis in a patient with HIV and negative serum PLA2R antibodies successfully treated with rituximab and highlights the diagnostic difficulties in this setting. Several reported cases in in HIV associated MN have been described. Charu et al [22]. present a series of eleven patients with HIV associated membranous nephropathy of whom one patient with circulating serum PLA2R antibodies demonstrated a favourable response to rituximab. El Husseini et al. [4] reported a similar clinical case managed with ACTH gel who similarly demonstrated positive serum anti-PLA2 testing. Meng et al. [23] reported the successful treatment of HIV-associated membranous nephropathy treated with a modified Ponticelli regimen, though PLA2R testing was not performed. Most case reports of MN in the context of HIV focus on the initiation of ART [24], though, one saw a profound response to glucocorticoid therapy alone [25]. None have reported improvement with rituximab in the context of negative serum anti PLA2R. A recent retrospective publication by Nikolopoulou *et. al.* [26] examined biopsy positivity in viral-associated MN cases where 4/6 HIV-associated MN cases demonstrated either PLA2R or THSD7A positivity and 3/4 HCV positive cases demonstrated PLA2R positivity. The single patient in this series with HIV and HCV coinfection was PLA2R negative on biopsy. This concords with findings in Charu et al. with 50% of HIV-associated MN cases demonstrating PLA2R biopsy positivity.

Readily available serum PLA2R testing has led to the proposal for the non-invasive diagnosis of MN [27]; however, this case demonstrates a clear discrepancy between serum and glomerular findings. A 2015 meta-analysis comparing serum autoantibody testing with glomerular staining across 19 trials [28] demonstrated a sensitivity of 68% for serum anti-PLA2R versus 78% for glomerular staining with a subsequent update by different authors in 2018 [29] demonstrating sensitivities of 65 and 79% respectively. It is recognised that serum PLA2R antibodies correlate with disease course in primary MN [30] and that serum antibodies are substantially more predictive of clinical course than the presence of glomerular staining [31]. Given the lower sensitivity of serum PLA2R testing, it remains an open question whether this alone is sufficient for diagnosis in suspected cases of MN [32].

The brisk response to rituximab implied an antibody mediated cause to this patient’s MN regardless of the negative serum testing. In PLA2R positive MN, it has been well demonstrated that rituximab treatment effectively lowers PLA2R titre [33]; this finding was seen in both the MENTOR and GEMRITUX trials [18, 34]. Similar suppression was seen for a patient with THSD7A positive disease in the MENTOR trial. Rituximab was also shown to benefit the antibody negative subgroups who may have had antibody positive disease that was not otherwise detected. It is possible that serum PLA2R was present, but below detectable levels using our assay, as there are differing sensitivities between laboratory techniques [35] with our laboratory utilising a commercial ELISA based technique. Initially serum PLA2R negative patients have been demonstrated to undergo ‘seroconversion’ many months into their disease process [36] and despite serial testing it is possible this would have occurred with further observation. Testing for THSD7A, NELL-1 and semaphorin 3b were not available at the time of this case report so their presence also cannot be excluded. Finally, it cannot be excluded that the improvement in proteinuria in this patient represented spontaneous remission. Spontaneous remission rates can be as high as 32% in a pooled cohort [37] and antibody-negative primary MN may be even higher [38]. Considering this, immunosuppressive therapy of any kind in those with HIV carries an infectious risk and any treatment must be balanced against this. Rituximab use in the context of chemotherapy for HIV associated lymphoma appears safe in those with CD4^+^ counts above 50cells/μL [39, 40].

Nephrotic syndrome more generally in the context of HIV infection has a broad differential including HIVAN, IgA nephropathy and HIV associated immune complex kidney disease (HIVICK), with relative prevalence shifting away from HIVAN in more recent studies [11, 41, 42]. HIVICK itself encompasses multiple pathological entities and definitions range from GN with strict ‘lupus like’ inclusions [43] to broader definitions encompassing multiple GN patterns [44, 45]. As such, varying pathophysiological mechanisms are proposed. A large trial examining clinical characteristics of broadly defined HIVICK found that those with MN on renal biopsy had on average more mild proteinuria [44]. HIVICK is significantly less likely to progress to end stage kidney disease (ESKD) than HIVAN and is more likely to be associated with lower viral loads and HAART exposure [45]. Our patient’s HIV had been well-controlled on a stable regimen for over 10 years, so is an unlikely contributor. Haemophilia itself is not known to be associated with MN, though rare cases have been seen in children with haemophilia B receiving immune tolerance induction therapy [46, 47].

We demonstrate the successful treatment of primary MN with rituximab in a patient with haemophilia with multiple stable viral comorbidities. This case highlights both the importance of obtaining a histological diagnosis in complex cases of MN as well as supports the use of rituximab as an immunomodulatory agent in this cohort of patients.

## Data Availability

Data sharing is not applicable to this article as no datasets were generated or analysed.

## References

[1] Esposito P, Rampino T, Gregorini M, Fasoli G, Gamba G, Dal Canton A (2013). Renal diseases in haemophilic patients: pathogenesis and clinical management. Eur J Haematol.

[2] Mazepa MA, Monahan PE, Baker JR, Riske BK, Soucie JM, Network USHTC (2016). Men with severe hemophilia in the United States: birth cohort analysis of a large national database. Blood.

[3] Hogan JJ, Mocanu M, Berns JS (2016). The native kidney biopsy: update and evidence for best practice. Clin J Am Soc Nephrol.

[4] El-Husseini A, Saxon D, Jennings S, Cornea V, Beck L, Sawaya BP (2016). Idiopathic membranous nephropathy: diagnostic and therapeutic challenges. Am J Nephrol.

[5] Althaf MM, Hussein MH, Abdelsalam MS, Amer SM (2014). Acute kidney injury in a diabetic haemophiliac: one step at a time. BMJ Case Rep.

[6] Kobayashi I, Ishimura E, Hirowatari K, Tsuchida T, Nishihira A, Shima H, Shidara K, Mori K, Inaba M, Wakasa K (2009). Renal biopsy in a patient with haemophilia a and cryoglobulinaemic membranoproliferative glomerulonephritis associated with hepatitis C virus infection. NDT Plus.

[7] Fogo AB, Lusco MA, Najafian B, Alpers CE (2015). AJKD atlas of renal pathology: membranous nephropathy. Am J Kidney Dis.

[8] Beck LH (2017). PLA2R and THSD7A: disparate paths to the same disease?. J Am Soc Nephrol.

[9] Sethi S, Debiec H, Madden B, Charlesworth MC, Morelle J, Gross L, Ravindran A, Buob D, Jadoul M, Fervenza FC (2020). Neural epidermal growth factor-like 1 protein (NELL-1) associated membranous nephropathy. Kidney Int.

[10] Sethi S, Debiec H, Madden B, Vivarelli M, Charlesworth MC, Ravindran A, Gross L, Ulinski T, Buob D, Tran CL (2020). Semaphorin 3B-associated membranous nephropathy is a distinct type of disease predominantly present in pediatric patients. Kidney Int.

[11] Szczech LA, Gupta SK, Habash R, Guasch A, Kalayjian R, Appel R, Fields TA, Svetkey LP, Flanagan KH, Klotman PE (2004). The clinical epidemiology and course of the spectrum of renal diseases associated with HIV infection. Kidney Int.

[12] Uchiyama-Tanaka Y, Mori Y, Kishimoto N, Nose A, Kijima Y, Nagata T, Umeda Y, Masaki H, Matsubara H, Iwasaka T (2004). Membranous glomerulonephritis associated with hepatitis C virus infection: case report and literature review. Clin Nephrol.

[13] Morales JM, Pascual-Capdevila J, Campistol JM, Fernandez-Zatarain G, Munoz MA, Andres A, Praga M, Martinez MA, Usera G, Fuertes A (1997). Membranous glomerulonephritis associated with hepatitis C virus infection in renal transplant patients. Transplantation.

[14] Alexander MP, Larsen CP, Gibson IW, Nasr SH, Sethi S, Fidler ME, Raissian Y, Takahashi N, Chari S, Smyrk TC (2013). Membranous glomerulonephritis is a manifestation of IgG4-related disease. Kidney Int.

[15] Larsen CP, Messias NC, Silva FG, Messias E, Walker PD (2013). Determination of primary versus secondary membranous glomerulopathy utilizing phospholipase A2 receptor staining in renal biopsies. Mod Pathol.

[16] Davenport A, Maciver AG, Hall CL, MacKenzie JC (1994). Do mesangial immune complex deposits affect the renal prognosis in membranous glomerulonephritis?. Clin Nephrol.

[17] Bomback AS, Fervenza FC (2018). Membranous nephropathy: approaches to treatment. Am J Nephrol.

[18] Fervenza FC, Appel GB, Barbour SJ, Rovin BH, Lafayette RA, Aslam N, Jefferson JA, Gipson PE, Rizk DV, Sedor JR (2019). Rituximab or cyclosporine in the treatment of membranous nephropathy. N Engl J Med.

[19] Praga M, Barrio V, Juarez GF, Luno J (2007). Grupo Espanol de Estudio de la Nefropatia M: tacrolimus monotherapy in membranous nephropathy: a randomized controlled trial. Kidney Int.

[20] Cattran DC, Appel GB, Hebert LA, Hunsicker LG, Pohl MA, Hoy WE, Maxwell DR, Kunis CL (2001). North America nephrotic syndrome study G: cyclosporine in patients with steroid-resistant membranous nephropathy: a randomized trial. Kidney Int.

[21] Ponticelli C, Altieri P, Scolari F, Passerini P, Roccatello D, Cesana B, Melis P, Valzorio B, Sasdelli M, Pasquali S (1998). A randomized study comparing methylprednisolone plus chlorambucil versus methylprednisolone plus cyclophosphamide in idiopathic membranous nephropathy. J Am Soc Nephrol.

[22] Charu V, Andeen N, Walavalkar V, Lapasia J, Kim JY, Lin A, Sibley R, Higgins J, Troxell M, Kambham N (2020). Membranous nephropathy in patients with HIV: a report of 11 cases. BMC Nephrol.

[23] Meng C, Pereira L, Guedes L, Nunes A, Pereira P (2018). Membranous nephropathy successfully treated with a Ponticelli regimen in a patient with HIV: do not assume that well-known secondary cause is the real cause!. Port J Nephrol Hypert.

[24] Aydin S, Mete B, Yilmaz M, Yenidunya G, Zaras R, Tunckale A, Tabak F (2012). A patient with HIV infection presenting with diffuse membranous glomerulonephritis in a country with a low HIV prevalence--remarkable remission with therapy. J Infect Public Health.

[25] Mattana J, Siegal FP, Schwarzwald E, Molho L, Sankaran RT, Gooneratne R, Ahuja TS, Singhal PC (1997). AIDS-associated membranous nephropathy with advanced renal failure: response to prednisone. Am J Kidney Dis.

[26] Nikolopoulou A, Teixeira C, Cook H, Roufosse C, Cairns T, Levy J, Pusey C, Griffith M (2020). Membranous nephropathy associated with viral infection. Clin Kidney J.

[27] Bobart SA, De Vriese AS, Pawar AS, Zand L, Sethi S, Giesen C, Lieske JC, Fervenza FC (2019). Noninvasive diagnosis of primary membranous nephropathy using phospholipase A2 receptor antibodies. Kidney Int.

[28] Dai H, Zhang H, He Y (2015). Diagnostic accuracy of PLA2R autoantibodies and glomerular staining for the differentiation of idiopathic and secondary membranous nephropathy: an updated meta-analysis. Sci Rep.

[29] Li W, Zhao Y, Fu P (2018). Diagnostic test accuracy of serum anti-PLA2R autoantibodies and glomerular PLA2R antigen for diagnosing idiopathic membranous nephropathy: an updated meta-analysis. Front Med (Lausanne).

[30] Ruggenenti P, Debiec H, Ruggiero B, Chianca A, Pelle T, Gaspari F, Suardi F, Gagliardini E, Orisio S, Benigni A (2015). Anti-phospholipase A2 receptor antibody titer predicts post-rituximab outcome of membranous nephropathy. J Am Soc Nephrol.

[31] Dong D, Fan TT, Wang YY, Zhang L, Song L, Zhang L (2019). Relationship between renal tissues phospholipase A2 receptor and its serum antibody and clinical condition and prognosis of idiopathic membranous nephropathy: a meta-analysis. BMC Nephrol.

[32] van de Logt AE, Fresquet M, Wetzels JF, Brenchley P (2019). The anti-PLA2R antibody in membranous nephropathy: what we know and what remains a decade after its discovery. Kidney Int.

[33] Beck LH, Fervenza FC, Beck DM, Bonegio RG, Malik FA, Erickson SB, Cosio FG, Cattran DC, Salant DJ (2011). Rituximab-induced depletion of anti-PLA2R autoantibodies predicts response in membranous nephropathy. J Am Soc Nephrol.

[34] Dahan K, Debiec H, Plaisier E, Cachanado M, Rousseau A, Wakselman L, Michel PA, Mihout F, Dussol B, Matignon M (2017). Rituximab for severe membranous nephropathy: a 6-month trial with extended follow-up. J Am Soc Nephrol.

[35] Katsumata Y, Okamoto Y, Moriyama T, Moriyama R, Kawamoto M, Hanaoka M, Uchida K, Nitta K, Harigai M (2020). Clinical usefulness of anti-M-type phospholipase-A-receptor antibodies in patients with membranous nephropathy and the comparison of three quantification methods. Immunol Med.

[36] van de Logt AE, Hofstra JM, Wetzels JF (2015). Serum anti-PLA2R antibodies can be initially absent in idiopathic membranous nephropathy: seroconversion after prolonged follow-up. Kidney Int.

[37] Polanco N, Gutierrez E, Covarsi A, Ariza F, Carreno A, Vigil A, Baltar J, Fernandez-Fresnedo G, Martin C, Pons S (2010). Spontaneous remission of nephrotic syndrome in idiopathic membranous nephropathy. J Am Soc Nephrol.

[38] Hoxha E, Harendza S, Pinnschmidt HO, Tomas NM, Helmchen U, Panzer U, Stahl RA (2015). Spontaneous remission of proteinuria is a frequent event in phospholipase A2 receptor antibody-negative patients with membranous nephropathy. Nephrol Dial Transplant.

[39] Sparano JA, Lee JY, Kaplan LD, Levine AM, Ramos JC, Ambinder RF, Wachsman W, Aboulafia D, Noy A, Henry DH (2010). Rituximab plus concurrent infusional EPOCH chemotherapy is highly effective in HIV-associated B-cell non-Hodgkin lymphoma. Blood.

[40] Spina M, Tirelli U (2005). Rituximab for HIV-associated lymphoma: weighing the benefits and risks. Curr Opin Oncol.

[41] Wearne N, Swanepoel CR, Boulle A, Duffield MS, Rayner BL (2012). The spectrum of renal histologies seen in HIV with outcomes, prognostic indicators and clinical correlations. Nephrol Dial Transplant.

[42] Kudose S, Santoriello D, Bomback AS, Stokes MB, Batal I, Markowitz GS, Wyatt CM, D'Agati VD (2020). The spectrum of kidney biopsy findings in HIV-infected patients in the modern era. Kidney Int.

[43] Fogo AB, Lusco MA, Najafian B, Alpers CE (2016). AJKD atlas of renal pathology: HIV-associated immune complex kidney disease (HIVICK). Am J Kidney Dis.

[44] Booth JW, Hamzah L, Jose S, Horsfield C, O'Donnell P, McAdoo S, Kumar EA, Turner-Stokes T, Khatib N, Das P (2016). Clinical characteristics and outcomes of HIV-associated immune complex kidney disease. Nephrol Dial Transplant.

[45] Foy MC, Estrella MM, Lucas GM, Tahir F, Fine DM, Moore RD, Atta MG (2013). Comparison of risk factors and outcomes in HIV immune complex kidney disease and HIV-associated nephropathy. Clin J Am Soc Nephrol.

[46] Verghese P, Darrow S, Kurth MH, Reed RC, Kim Y, Kearney S (2013). Successful management of factor IX inhibitor-associated nephrotic syndrome in a hemophilia B patient. Pediatr Nephrol.

[47] Dharnidharka VR, Takemoto C, Ewenstein BM, Rosen S, Harris HW (1998). Membranous glomerulonephritis and nephrosis post factor IX infusions in hemophilia B. Pediatr Nephrol.

